# Emerging trends and hotspots in lung cancer-prediction models research

**DOI:** 10.1097/MS9.0000000000002648

**Published:** 2024-10-18

**Authors:** Qiong Ma, Hua Jiang, Shiyan Tan, Fengming You, Chuan Zheng, Qian Wang, Yifeng Ren

**Affiliations:** a Hospital of Chengdu University of Traditional Chinese Medicine, Chengdu, Sichuan Province, China; b TCM Regulating Metabolic Diseases Key Laboratory of Sichuan Province, Hospital of Chengdu University of Traditional Chinese Medicine, Chengdu, Sichuan Province, China

**Keywords:** bibliometrics, lung cancer-prediction models, prognosis, pulmonary nodule, worldwide trends

## Abstract

**Objective:**

In recent years, lung cancer-prediction models have become popular. However, few bibliometric analyses have been performed in this field.

**Methods:**

This study aimed to reveal the scientific output and trends in lung cancer-prediction models from a global perspective. In this study, publications were retrieved and extracted from the Web of Science Core Collection (WoSCC) database. CiteSpace 6.1.R3 and VOSviewer 1.6.18 were used to analyze hotspots and theme trends.

**Results:**

A marked increase in the number of publications related to lung cancer-prediction models was observed. A total of 2711 institutions from in 64 countries/regions published 2139 documents in 566 academic journals. China and the United States were the leading country in the field of lung cancer-prediction models. The institutions represented by Fudan University had significant academic influence in the field. Analysis of keywords revealed that lncRNA, tumor microenvironment, immune, cancer statistics, The Cancer Genome Atlas, nomogram, and machine learning were the current focus of research in lung cancer-prediction models.

**Conclusions:**

Over the last two decades, research on risk-prediction models for lung cancer has attracted increasing attention. Prognosis, machine learning, and multi-omics technologies are both current hotspots and future trends in this field. In the future, in-depth explorations using different omics should increase the sensitivity and accuracy of lung cancer-prediction models and reduce the global burden of lung cancer.

## Graphical abstract

**Figure:**
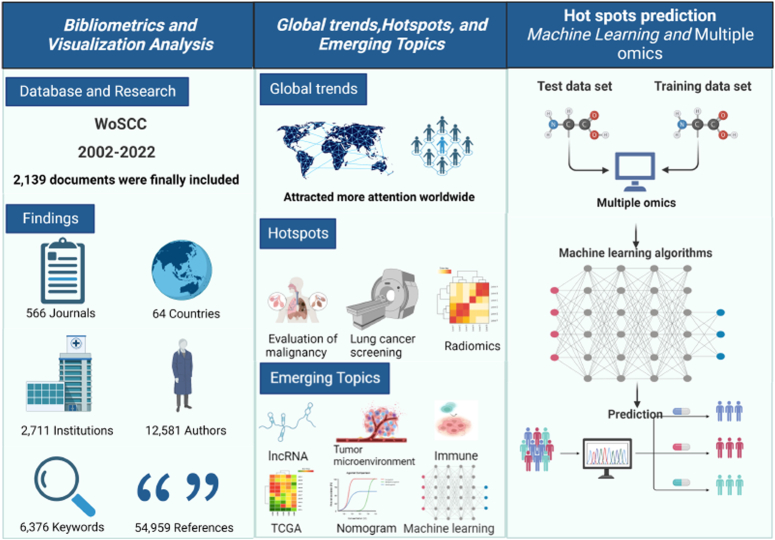

## Introduction

Highlights

- This cross-sectional study aimed to reveal the scientific output and trends in lung cancer-prediction models from a global perspective.
- Prognosis, machine learning, and multi-omics technologies are both current hotspots and future trends in this field.
- In future, in-depth explorations using different omics should increase the sensitivity and accuracy of lung cancer-prediction models and reduce the global burden of lung cancer.

Lung cancer remains the deadliest and second most common cancer worldwide,^1–3^ and early detection remains challenging.^4^ Despite treatment advances, advanced lung cancer patients continue to have unfavorable prognoses.^5–7^ Clinical survival outcomes are strongly associated with the disease stage.^8,9^ Earlier diagnosis allows the 5-year relative survival to increase from 6% for distant-stage disease to 33% for regional-stage disease and 60% for localized-stage disease.^2^ Thus, early detection and treatment are the most effective strategies for reducing lung cancer-associated mortality and economic burden.^5^

With advances in cancer screening techniques,^10^ particularly improved low-dose computed tomography (LDCT) resolution,^11,12^ hundreds of thousands of patients are diagnosed with pulmonary nodules annually.^13^ Due to a significant false-positive rate and increased risk of overdiagnosis, numerous patients with pulmonary nodules undergo unnecessary procedures.^3,12,14^ However, pulmonary nodule or lung cancer risk-prediction models can significantly reduce the false-positive rate in lung cancer screening. Some current guidelines recommend the use of prediction models for lung cancer screening.^15^ For example, the National Comprehensive Cancer Network guidelines for lung cancer screening endorse risk-prediction model utilization to identify high-risk individuals.^16^

Initially, lung cancer-prediction models were based primarily on patients’ CT characteristics and clinical information.^17–19^ With widespread omission of other important biomarkers and patient characteristics, the false-positive, overdiagnosis, and unnecessary treatment rates were markedly high.^20,21^ Various traditional and deep-learning models based on clinical, epidemiological factors and multi-omics methods (including radiomics, genomics, proteomics, and metabolomics) were developed to improve the accuracy and sensitivity of lung cancer-prediction models.^22–24^

The number of lung cancer-prediction models is growing; however, these studies have not been systematically measured. Although there have been some reviews on lung cancer risk-prediction models with different emphases,^22,25^ a comprehensive and visualized analysis of the evolution and trends of these models is still lacking. Thus, in this study, we characterized the lung cancer risk-prediction model landscape and explored the trends in this field by using bibliometric analyses and sought to offer perspectives on future research directions.

## Materials and methods

### Data source and retrieval

In this study, the literature search and data download from the Web of Science Core Collection database (WoSCC) were completed in a single day (26 October 2022).

Search strategy was as Supplementary Table S1, Supplemental Digital Content 1, http://links.lww.com/MS9/A614. Publications (articles and reviews) in English, from 1 January 2002, to 26 October 2022, were included. Search results, as “Full Record and Cited References,” were exported as “Plain Text Files” and stored in “download.txt” format. The exported data included the number of publications, the number of citations, journals, countries, institutions, authors, keywords, and references.

The published articles or reviews about the lung cancer-prediction models were included. Exclusion criteria were: conference abstracts, unpublished articles, repeated publications, corrigendum documents, dissertations, letters, unrelated articles.

Two authors (Q.M. and H.J.) independently assessed documents for eligibility. Disagreements were resolved by discussion to consensus with a third author (Y.F.R.). Informed consent and ethical approval were not required for this type of study.^26^

### Bibliometrics and visualization analysis

The dataset was exported to bibliometric analysis software (VOSviewer 1.6.18,^27^ CiteSpace 6.1. R3,^28,29^ and the Online Analysis Platform of Bibliometrics http://bibliometric.com/) to analyze the trends and emerging foci of lung cancer-prediction models over the past 20 years.^30^ The impact factor (IF) of these journals was determined by Journal Citation Reports (JCR) 2021.

The annual numbers of documents and citations were exported to Microsoft Excel 2020 to statistics and visual analysis. We created a linear graph to reveal the annual growth in the numbers of documents and citations. We performed mathematical function fitting for the curves of documents.^31^

The co-authorship network of authors, countries, and institutions, co-citation network of journals and references, and co-occurrence network of keywords were identified using VOSviewer 1.6.18.^32,33^ In co-occurrence networks, the size of the node indicates the frequency of occurrence and the density of links between nodes represents the intensity of cooperation.^34^ Chord diagrams were made using Chartculator (https://charticulator.com/), the width of the links represent the strength of the cooperative for different countries. The keywords cluster analysis was using VOSviewer 1.6.18. High-frequency co-cited references were analysis by CiteSpace 6.1. R3.

The node label presents the keyword, links connecting two nodes indicate a co-occurrence relationship between two keywords, and link clusters represent keyword cooperation relationships.^35^ To explore future research focus points and trends, we used CiteSpace 6.1.R3 for the timeline and burst detection of keywords and references.

## Results

### Search results

**Figure 1:**
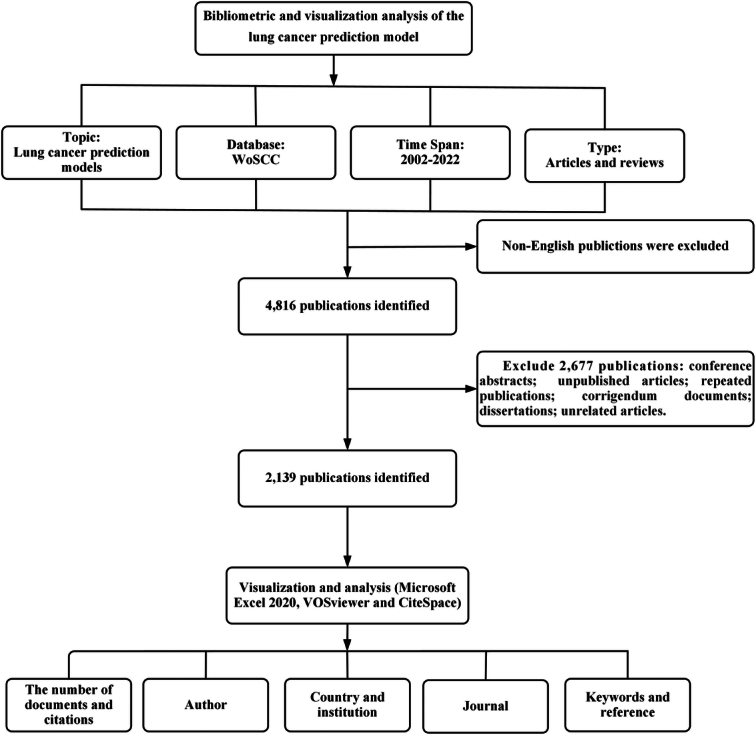
Flow diagram of study selection of lung cancer-prediction model research.

A total of 4816 publications on the topic of the “lung cancer-prediction model” were obtained from the WoSCC. After screening the titles, abstracts, and full texts of the papers, 2139 documents were included (Fig. 1).

### Annual growth trend of publication outputs

**Figure 2:**
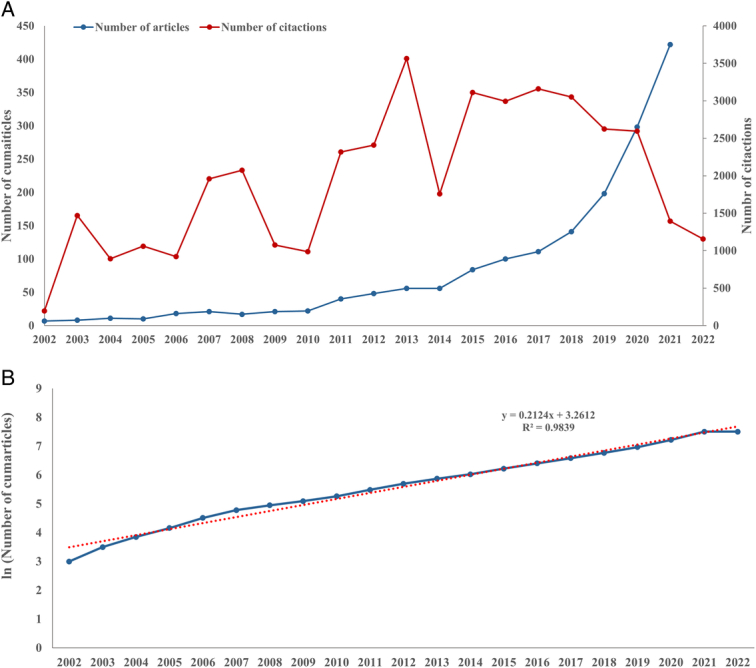
(A) The annual number of documents and citations on lung cancer-prediction models from 2002 to 2022; (B) The annual number of documents on lung cancer-prediction models and the curve fitting of publications.

The 2139 documents, including 1892 articles (88.45%) and 247 reviews (11.56%), were analyzed. The annual growth trends of documents and citations are shown (Fig. 2A). The annual number of publications increased over time, with fluctuations in 2010 and 2014. Relatively few studies were published during 2002–2015 (*n*=419), but the number increased rapidly thereafter, reaching 1720 (80.41%) during 2016–2022. The number of papers published in 2022 alone was 450, indicating increased attention to this field. Curve fitting showed that the trend leveled off (Fig. 2B). The results showed the researches of lung cancer-prediction models were growing steadily.

Early articles were cited relatively few times, as the field was in the early stages of development. The trend in the number of citations over time exhibited relatively large fluctuations. The number of citations began to surge in 2011, rising by a factor of three during 2011–2013, and peaking in 2013 (*n*=3564). Notably, the number of publications has increased steadily every year, but the number of citations has decreased during 2015–2022.

### Journal distribution

**Figure 3:**
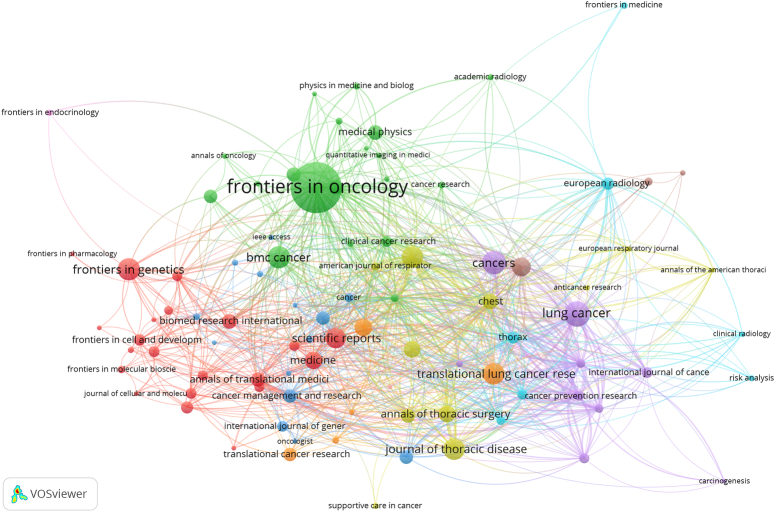
The co-authorship network of journals related to lung cancer-prediction model research.

The retrieved articles had been published in 566 journals. Table 1 lists the top-20 most involved journals. The top-20 journals published 703 articles on lung cancer-prediction models, accounting for 32.87% of all articles. The most productive journal was *Frontiers in Oncology* (120 publications, 435 citations), followed by *Lung Cancer* (47 publications, 763 citations), and *Cancers* (42 publications, 194 citations). Among the top-20 academic journals, the journal with the highest IF, that is the *Journal of Thoracic Oncology* (IF=20.121), was from the USA. In total, 2139 articles were cited 39829 times. Documents published in *Chest* were cited most (2077). When the minimum number of documents to five in VOSviewer, the co-occurrence network of the journals had 91 items, 10 clusters, and 863 links. *Frontiers in Oncology* published the most reports (Fig. 3). The largest cluster (in red), consisting of 21 journals, and *Frontiers in Genetics*, *Scientific Reports,* and *Annals of Translational Medicine* were center. Thus, most articles were published in authoritative journals, suggesting that lung cancer-prediction models have attracted the attention of scientists globally.

**Table 1:** The top-20 productive journals in the field of lung cancer-prediction model research

Rank	Journal	Documents	Country	Total link strength	Total citations	IF^a^	Quartile citation category
1	*Frontiers In Oncology*	120	Switzerland	175	435	5.738	Q2
2	*Lung Cancer*	47	Netherlands	198	763	6.081	Q2
3	*Cancers*	42	Switzerland	86	194	6.575	Q1
4	*BMC CANCER*	39	the UK	55	325	4.638	Q2
5	*Frontiers In Genetics*	39	Switzerland	44	88	4.772	Q1
6	*Journal Of Thoracic Oncology*	37	the USA	196	1268	20.121	Q1
7	*Journal Of Thoracic Disease*	37	China	87	222	3.005	Q3
8	*Translational Lung Cancer Research*	37	China	87	183	4.726	Q2
9	*Scientific Reports*	34	the UK	68	360	4.997	Q2
10	*Plos One*	31	the USA	87	1006	3.752	Q2
11	*Medicine*	28	the USA	40	258	1.817	Q3
12	*Thoracic Cancer*	28	China	68	192	3.223	Q3
13	*Annals Of Thoracic Surgery*	27	the USA	69	728	5.113	Q2
14	*European Journal Of Cardio-Thoracic Surgery*	27	Netherlands	45	457	4.534	Q2
15	*Chest*	23	the USA	192	2077	11.393	Q1
16	*Medical Physics*	22	the USA	23	391	4.506	Q2
17	*Biomed Research International*	22	the USA	33	124	3.246	Q3
18	*International Journal Of Radiation Oncology Biology Physics*	21	the USA	74	1541	8.013	Q1
19	*Radiotherapy And Oncology*	21	Germany	32	750	6.901	Q2
20	*Journal Of Thoracic And Cardiovascular Surgery*	21	the USA	78	536	6.439	Q2

a The impact factors (IF) of journals were obtained from the 2021 Web of Science Journal Citation Reports (JCR).

### Distribution and co-authorship of the countries/regions

Sixty-four countries/regions contributed to lung cancer-prediction model research. The top-20 most-productive countries are enumerated in Table 2. The vast majority of publications came from China (1125 publications, 9653 citations) and the USA (556 publications, 19182 citations), followed by the UK (127 publications, 4655 citations), Canada (105 publications, 3848 citations), and the Netherlands (101 publications, 4268 citations). The top-20 countries in terms of the number of reports included 13 European countries, 4 Asian countries, 2 North American countries, and 1 Oceanic country.

**Table 2:** The top-20 productive countries in the field of lung cancer-prediction model research

Rank	Countries	Documents	Percentage (%)	Total link strength	Total citations
1	China	1125	52.59	234	9653
2	the USA	556	25.99	478	19182
3	the UK	127	5.94	232	4655
4	Canada	105	4.91	190	3848
5	Netherlands	101	4.72	203	4268
6	Italy	94	4.39	195	4227
7	Germany	87	4.07	187	2503
8	Japan	82	3.83	42	1179
9	France	70	3.27	180	2386
10	Spain	59	2.76	138	1520
11	Australia	58	2.71	108	1390
12	South Korea	57	2.66	20	920
13	Denmark	36	1.68	128	1442
14	Belgium	28	1.31	70	1031
15	Norway	24	1.12	106	870
16	India	24	1.12	9	364
17	Sweden	22	1.03	91	448
18	Austria	18	0.84	74	701
19	Switzerland	17	0.79	52	771
20	Poland	15	0.70	50	695

**Figure 4:**
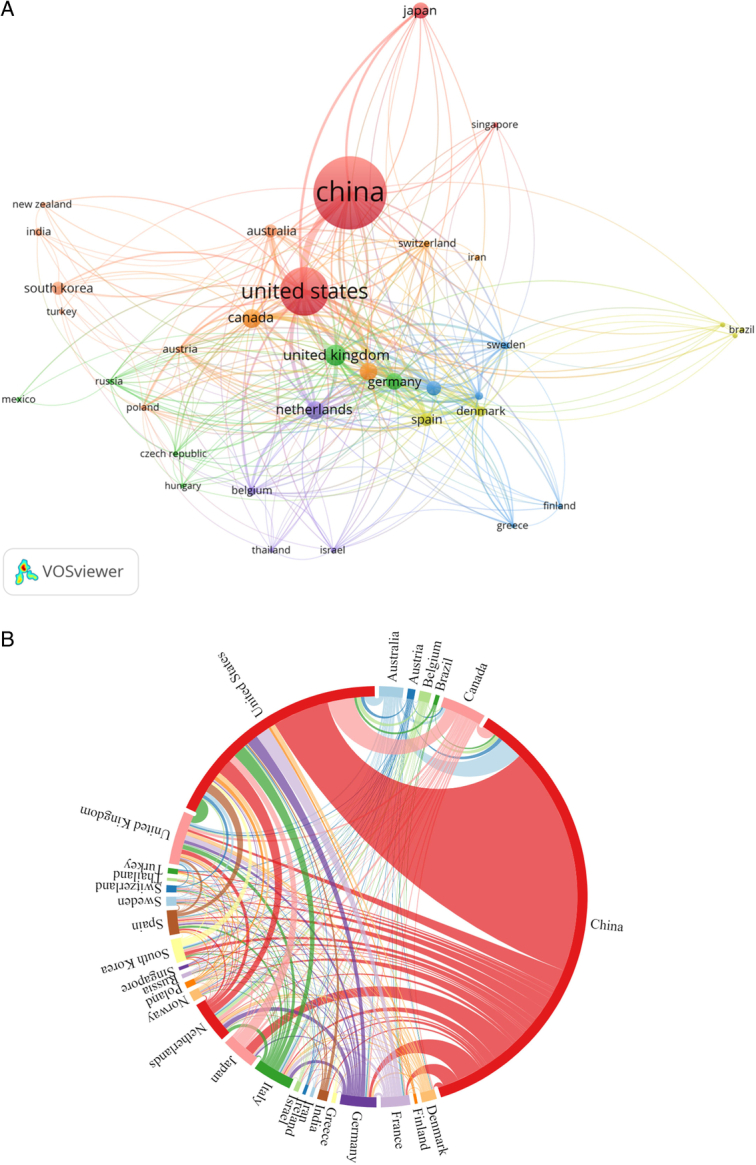
(A) The co-authorship network of countries/regions related to lung cancer-prediction model research; (B) Chord diagrams of collaborations among countries/regions related to lung cancer-prediction model research.

In VOSviewer, we set the minimum number of documents to five to show the co-authorship analysis of countries, and a geographical origin-based analysis of the retrieved documents was mapped. It containing 35 items, 7 clusters, and 294 links (Fig. 4A). The largest cluster (in orange) consisted of seven countries, centric Australia, South Korea, and India. The USA had the most cooperative partners (*n*=31), followed by the UK (*n*=27), Italy (*n*=27), Germany (*n*=27), Spain (*n*=27), and Denmark (*n*=27). The collaboration map indicated relatively close cooperation among countries/regions (Fig. 4B). China collaborated closely with the USA.

### Distribution and co-authorship of institutions

In total, 2711 institutions contributed to lung cancer-prediction model research. Table 3 enumerates the top-20 institutions. The most productive institution was Fudan University (60 publications, 1595 citations), followed by Peking Union Medical College (56 publications, 565 citations), and Zhejiang University (54 publications, 312 citations). The top-20 institutions included 15 institutions in China and 5 in the USA.

**Table 3:** The top-20 productive institutions in the field of lung cancer-prediction model research

Rank	Institutions	Countries	Documents	Total Link Strength	Total citations
1	Fudan University	China	60	79	1595
2	Peking Union Medical College	China	56	85	565
3	Zhejiang University	China	54	63	312
4	Nanjing Medical University	the USA	53	69	444
5	The University of Texas MD Anderson Cancer Center	China	48	72	1010
6	Shandong University	China	46	43	721
7	Shanghai Jiao Tong University	China	45	70	316
8	Tongji University	China	41	45	994
9	Sun Yat Sen University	the USA	41	76	581
10	Memorial Sloan Kettering Cancer Center	China	40	61	1187
11	Huazhong University of Science and Technology	China	36	39	290
12	National Cancer Institute	the USA	35	99	2682
13	Sichuan University	China	35	45	580
14	Chinese Academy of Sciences	China	35	69	535
15	Peking University	China	35	37	418
16	Capital Medical University	the USA	34	38	218
17	Brock University	Canada	31	121	1763
18	Southern Medical University	the USA	30	44	208
19	Central South University	China	30	10	156
20	Wuhan University	China	28	20	149

**Figure 5:**
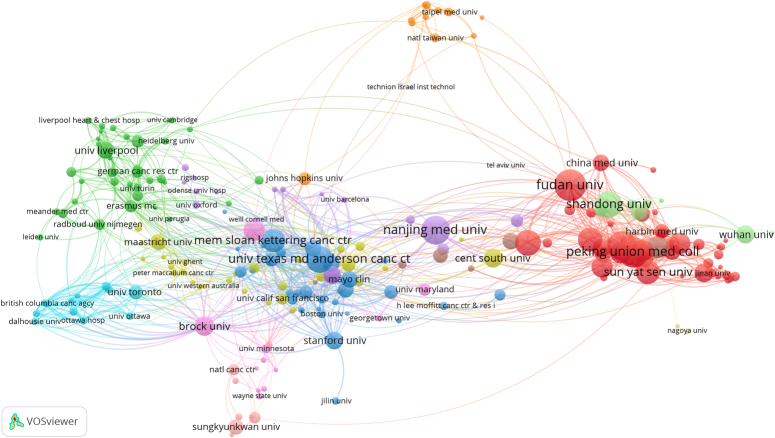
The co-authorship network of institutions related to lung cancer-prediction model research.

Figure 5 shows a collaborative network for institutions with a minimum of 10 publications, included 102 items, 7 clusters, and 802 links. The red cluster, consisting of 32 institutions centered on Fudan University, Shanghai Jiao Tong University, Sun Yat Sen University, and Peking Union Medical College, was the largest cluster. Harvard School of Medicine had the most cooperating partners (*n*=34), followed by Shanghai Jiao Tong University (*n*=33), Sun Yat Sen University (*n*=32), and Harvard University (*n*=32).

### Distribution and co-authorship of individuals

**Figure 6:**
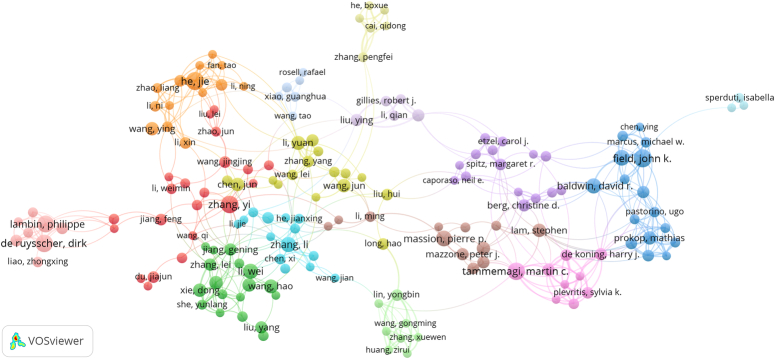
The co-authorship network of authors related to lung cancer-prediction model research.

The total number of authors involved in lung cancer-prediction model publications was 12 581, of which 22 authors published more than 10 documents and 199 published more than 5 documents. On average, there are 5.88 authors per documents. The 20 most-productive authors are listed in Table 4. John K Fields (17 publications, 1211 citations) and Jie He (17 publications, 88 citations) published the most studies, followed by Dirk De Ruysscher (16 publications, 634 citations), Martin C Tammemagi (16 publications, 514 citations), and Yi Zhang (16 publications, 102 citations). When we set the minimum number of articles was five, the authors co-authorship network included 171 items, 15 clusters, and 517 links (Fig. 6). The red cluster, consisting of 21 authors and Yi Zhang, Feng Jiang, and Jingjing Wang were center of the cluster. The authors who collaborate most are Li Zhang (*n*=17), followed by Harry J de Koning (*n*=16), and Martin C Tammemagi (*n*=15).

**Table 4:** The top-20 productive authors in the field of lung cancer-prediction model research

Rank	Author	Documents	Total citations	Total link strength	Average citation per article
1	Field John K.	17	1211	45	71.24
2	He Jie	17	88	48	5.18
3	De Ruysscher Dirk	16	634	34	39.63
4	Tammemagi Martin C.	16	514	35	32.13
5	Zhang Yi	16	102	13	6.38
6	Lambin Philippe	15	747	34	49.8
7	Duffy Stephen W.	14	1032	41	73.71
8	Massion Pierre P.	14	472	14	33.71
9	Baldwin David R.	13	782	22	60.15
10	Li Wei	13	429	25	33
11	Zhang Li	13	221	9	17
12	Li Yuan	11	109	8	9.91
13	Wang Hao	11	66	20	6
14	Chen Chang	10	815	19	81.5
15	Chen Jun	10	327	38	32.7
16	Lam Stephen	10	203	12	20.3
17	Prokop Mathias	10	178	25	17.8
18	Silvestri Gerard A.	10	173	9	17.3
19	Wang Jun	10	152	25	15.2
20	Wang Ying	10	67	1	6.7

### Keyword co-occurrence, clusters, and burst

**Figure 7:**
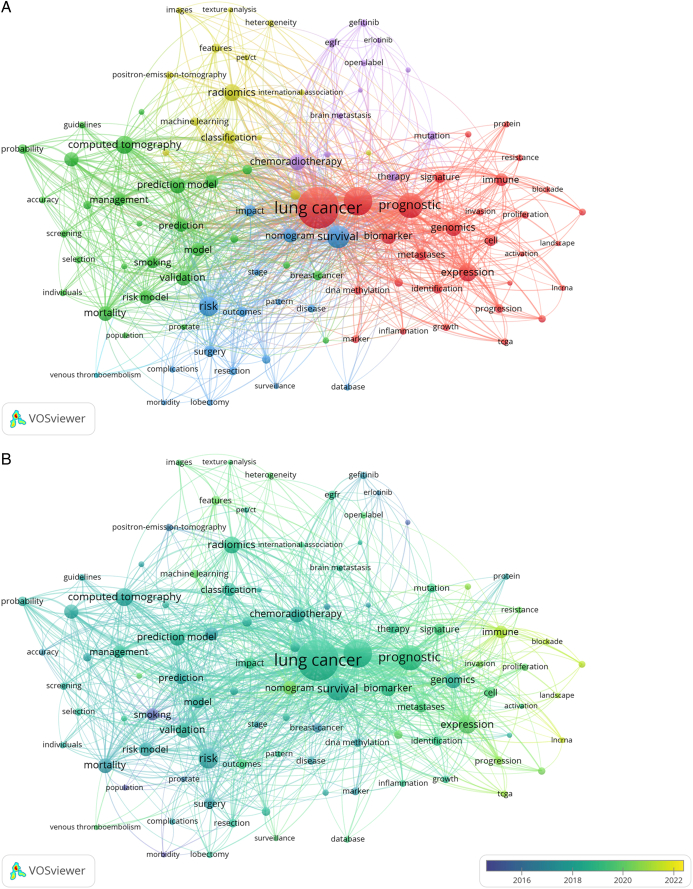
(A) The co-occurrence cluster analysis of the top 100 Keywords; (B) Overlay visualization map of co-occurring keywords.

The 2139 retrieved publications cited 6376 keywords. To explore research hotspots in this field, we created a visualization network map of co-occurrence keywords with the extraction frequencies of the top-100 keywords (Fig. 7A). It contained five clusters and 3290 links. The keywords lung cancer (1298), cancer (615), prognosis (495), survival (402), radiomics (305), computed tomography (251), and expression (251) were placed at the center of the network.

The top-100 keywords were classified into five different clusters by VOSviewer, as follows (Fig. 7A): cluster 1 “lung cancer” (red color) included lung cancer, cancer, prognostic, genomics, and expression; cluster 2 “prediction model” (green color) included computed tomography, pulmonary nodule, prediction model, risk model, and management; cluster 3 “survival” (blue color) included survival, risk, nomogram, and outcomes; cluster 4 “radiomics” (yellow color) included classification, features, machine learning, and heterogeneity; cluster 5 “therapy” (purple color) included chemoradiotherapy, erlotinib, gefitinib, mutation, and EGFR.

Keywords were colored based on their average appearing year (AAY) to explore evolutionary trends over time (Fig. 7B). The recently emerged keywords were “lncRNA” (AAY: 2021.30), “tumor microenvironment” (AAY: 2021.08), “immune” (AAY: 2020.88), “TCGA” (AAY: 2020.61), “cancer statistics” (AAY: 2020.50), “nomogram” (AAY: 2020.17), and “machine learning” (AAY: 2019.91).

**Figure 8:**
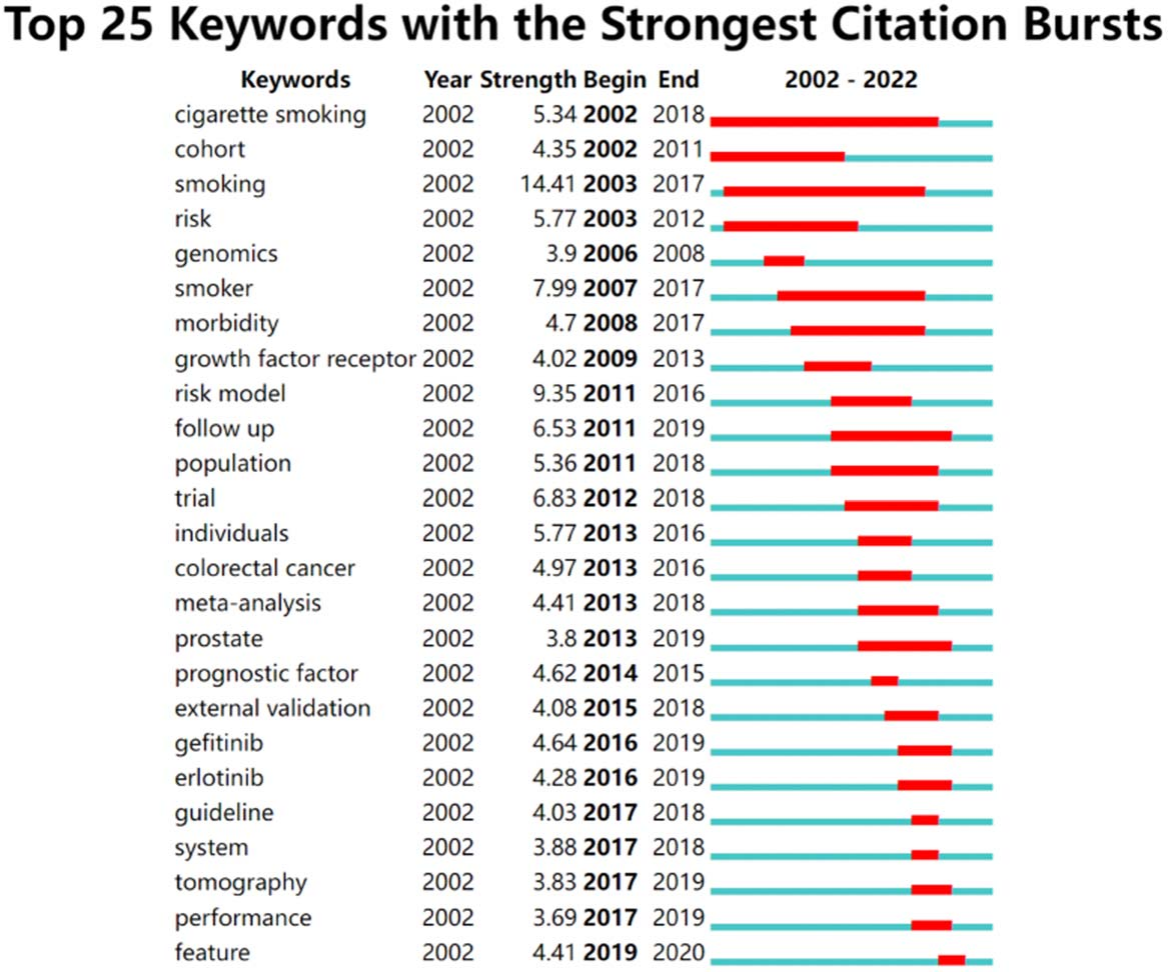
Top 25 keywords with the strongest citation bursts of lung cancer-prediction model.

The keyword “smoking” (14.41) had the strongest citation burst, followed by “risk models” (9.35), “smoker” (7.99), and “trail study” (6.83) in Figure 8. The most recent keywords with citation bursts that occurred in the past 5 years were “guideline,” “system,” “tomography,” “performance,” and “feature.”

### Co-cited references and reference burst

**Figure 9:**
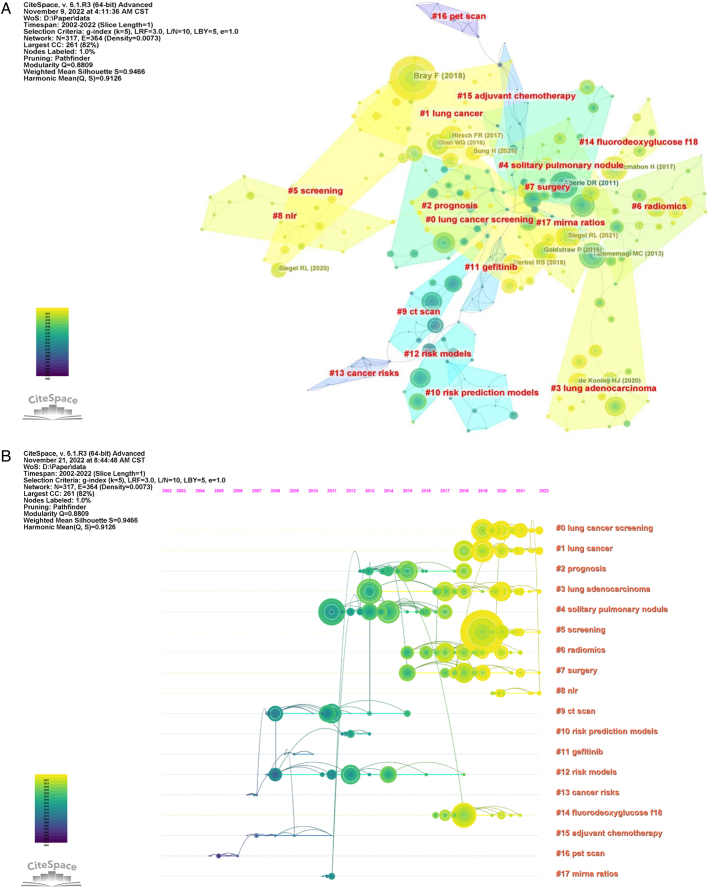
(A) Map of co-cited references in the field of lung cancer-prediction model research during 2002–2022; (B) Timeline and clustering view of all of co-cited references.

We listed the top-20 co-cited references (Table 5). From the table, most of the co-cited references came from top journals, such as *The New England Journal of Medicine, CA: A Cancer Journal for Clinicians*, and *Nature*. More than half of these studies were related to the epidemiology of lung cancer and prediction models. A review entitled “*Reduced lung-cancer mortality with low-dose computed tomographic screening*” by the National Lung Screening Trial Research Team,^36^ published in *The New England Journal of Medicine,* was cited most (*n*=291). They showed that screening with LDCT reduce significantly lung cancer-related mortality, emphasizing its importance in lung cancer screening and diagnosis. The 2139 retrieved publications cited 54959 references. We plotted the reference co-citation network and analyzed the research hotspots and trends using co-cited references. The literature shown in the map of the co-citation network was classified into 17 clusters using CiteSpace (Fig. 9A). In terms of distribution, the clustering was relatively concentrated, mainly including #0 lung cancer screening, #1 lung cancer, #2 prognosis, #3 lung adenocarcinoma, #4 solitary pulmonary nodule, #5 screening, and #6 radiomics. In addition, we constructed a timeline map (Fig. 9B). The results indicated that the field has developed rapidly over the past 10 years. It is noteworthy that #0 lung cancer screening, #1 lung cancer, #5 screening, #6 radiomics, #7 surgery, #8 NLR, and #14 fluorodeoxyglucose f18 analysis mainly arose during 2016–2022, indicating that these clusters were hotspots of lung cancer-prediction model research.

**Table 5:** The top-20 cited articles related to lung cancer-prediction model research

Rank	Citations	Total link strength	The title of article	Year	First author	Journal	Country	IF
1	291	2461	Reduced lung-cancer mortality with low-dose computed tomographic screening	2011	National Lung Screening Trial Research Team	*The New England journal of Medicine*	the USA	176.079
2	226	890	Global cancer statistics 2018: GLOBOCAN estimates of incidence and mortality worldwide for 36 cancers in 185 countries	2018	Freddie Bray	*CA: a cancer journal for clinicians*	the USA	286.130
3	132	1531	Selection criteria for lung-cancer screening	2013	Martin C Tammemägi	*The New England journal of Medicine*	the USA	176.079
4	116	1221	Probability of cancer in pulmonary nodules detected on first screening CT	2013	Annette McWilliams	*The New England journal of Medicine*	the USA	176.079
5	110	1346	The LLP risk model: an individual risk prediction model for lung cancer	2008	A Cassidy	*British Journal of Cancer*	the UK	9.082
6	106	1234	Variations in lung cancer risk among smokers	2003	Peter B Bach	*Journal of the National Cancer Institute*	the USA	11.816
7	100	863	The probability of malignancy in solitary pulmonary nodules. Application to small radiologically indeterminate nodules	1997	S. J. Swensen	*Archives of internal medicine*	the USA	1.2
8	96	1118	A risk model for prediction of lung cancer	2007	Margaret R Spitz	*Journal of the National Cancer Institute*	the USA	11.816
9	85	846	Screening for lung cancer: U.S. Preventive Services Task Force recommendation statement	2014	Virginia A Moyer	*Annals of Internal Medicine*	the USA	51.598
10	80	330	Tutorial in biostatistics multivariable prognostic models: issues in developing models, evaluating assumptions and adequacy, and measuring and reducing errors.	1996	FRANK E. HARRELL Jr	*Statistics in Medicine*	the UK	2.497
11	79	355	International association for the study of lung cancer/american thoracic society/european respiratory society international multidisciplinary classification of lung adenocarcinoma	2011	William D Travis	*Journal of Thoracic Oncology*	the USA	20.121
12	78	430	Robust enumeration of cell subsets from tissue expression profiles	2015	Aaron M Newman	*Nature Methods*	the USA	47.99
13	78	395	clusterProfiler: an R package for comparing biological themes among gene clusters	2012	Guangchuang Yu	*OMICS*	the USA	3.978
14	77	335	Cancer statistics in China, 2015	2016	Wanqing Chen	*CA: a cancer journal for clinicians*	the USA	286.130
15	75	380	The IASLC Lung Cancer Staging Project: Proposals for Revision of the TNM Stage Groupings in the Forthcoming (Eighth) Edition of the TNM Classification for Lung Cancer	2016	Peter Goldstraw	*Journal of Thoracic Oncology*	the USA	20.121
16	74	299	The biology and management of non-small cell lung cancer	2018	Roy S Herbst	*Nature*	the UK	69.504
17	74	966	Lung cancer risk prediction: Prostate, Lung, Colorectal And Ovarian Cancer Screening Trial models and validation	2011	C Martin Tammemagi	*Journal of the National Cancer Institute*	the USA	11.816
18	72	329	Decoding tumour phenotype by noninvasive imaging using a quantitative radiomics approach	2014	Hugo J W L Aerts	*Nature Communications*	the UK	17.694
19	71	662	A clinical model to estimate the pretest probability of lung cancer in patients with solitary pulmonary nodules	2007	Michael K Gould	*Chest*	the USA	11.393
20	70	352	limma powers differential expression analyses for RNA-sequencing and microarray studies	2015	Matthew E Ritchie	*Nucleic Acids Research*	the UK	19.160

CT, computed tomography; IF, impact factor; LLP, Liverpool Lung Project.

## Discussion

### Global trends in lung cancer-prediction model research

We identified 2139 documents published between 2002 and 2022 from 64 countries. Over the last two decades, the number of documents of lung cancer-prediction models has increased steadily every year, as high-throughput sequencing technology and artificial intelligence have evolved, whereas the number of citations exhibited large fluctuations.^37^ The low number of citations during 2002–2010 may have been related to the field being in its infancy. The number of citations decreased during 2015–2022, possibly because citations peak by 3–10 years after publication.^38^

A total of 12581 authors from 2711 institutions in 64 countries/regions published articles on lung cancer-prediction models, showing that the research has aroused global interest. China was the most productive country, while the documents of the USA had the highest number of citations. The USA was at the core of international cooperation, with strong connections to China, Canada, Japan, Australia, and Singapore. The top-20 institutions included 15 institutions in China and five in the USA. The number of documents from China has increased continuously in recent years, reflecting the high value it places on lung cancer-predictive model research.

Among individual authors, John K Field, from the Department of Molecular and Clinical Cancer Medicine of the University of Liverpool, ranked first, with 17 publications (1211 citations). He published an article “*Lung cancer screening with low dose computed tomography*” in *BMJ*, which showed that LDCT reduced lung cancer mortality in high-risk individual.^39^ Jie He, from the Chinese Academy of Medical Sciences, also published 17 documents, which were cited 88 times. Li Zhang, with 17 collaborations had the largest node size for collaboration. These findings emphasize the importance of collaboration among countries, institutions, and authors to advance this field.

### Research hotspots and emerging topics

To the best of our knowledge, no previous report provided a visual analysis of documents of lung cancer-prediction models using a bibliometric approach. Based on the reference co-citation and keyword analyses, we outlined the research hotspots and emerging topics in the field over the last two decades.

Keyword analysis revealed that prognosis, therapy, survival, and radiomics are current focus points of lung cancer-prediction model research. Notably, lncRNA (AAY: 2021.30), tumor microenvironment (AAY: 2021.08), immune (AAY: 2020.88), TCGA (AAY: 2020.61), cancer statistics (2020.50), nomogram (AAY: 2020.17), and machine learning (AAY: 2019.91) have appeared more frequently in recent years (Fig. 7B). With high-throughput sequencing technology have rapidly developing roles, an increasing number of models consider new predictors for diagnosis, including lncRNA, genomics, immunity, and radiomics.^40,41^ Furthermore, lung cancer-prediction models are becoming more accurate due to the rapid development of machine learning and nomograms.^42^

Lung cancer-prediction models have a history of 20 years. The most extensively used model was the Mayo Clinic model, established by Swensen *et al*.^43^ in 1997. With the popularization of lung cancer screening, more studies have demonstrated the potential of lung cancer-prediction models.

**Figure 10:**
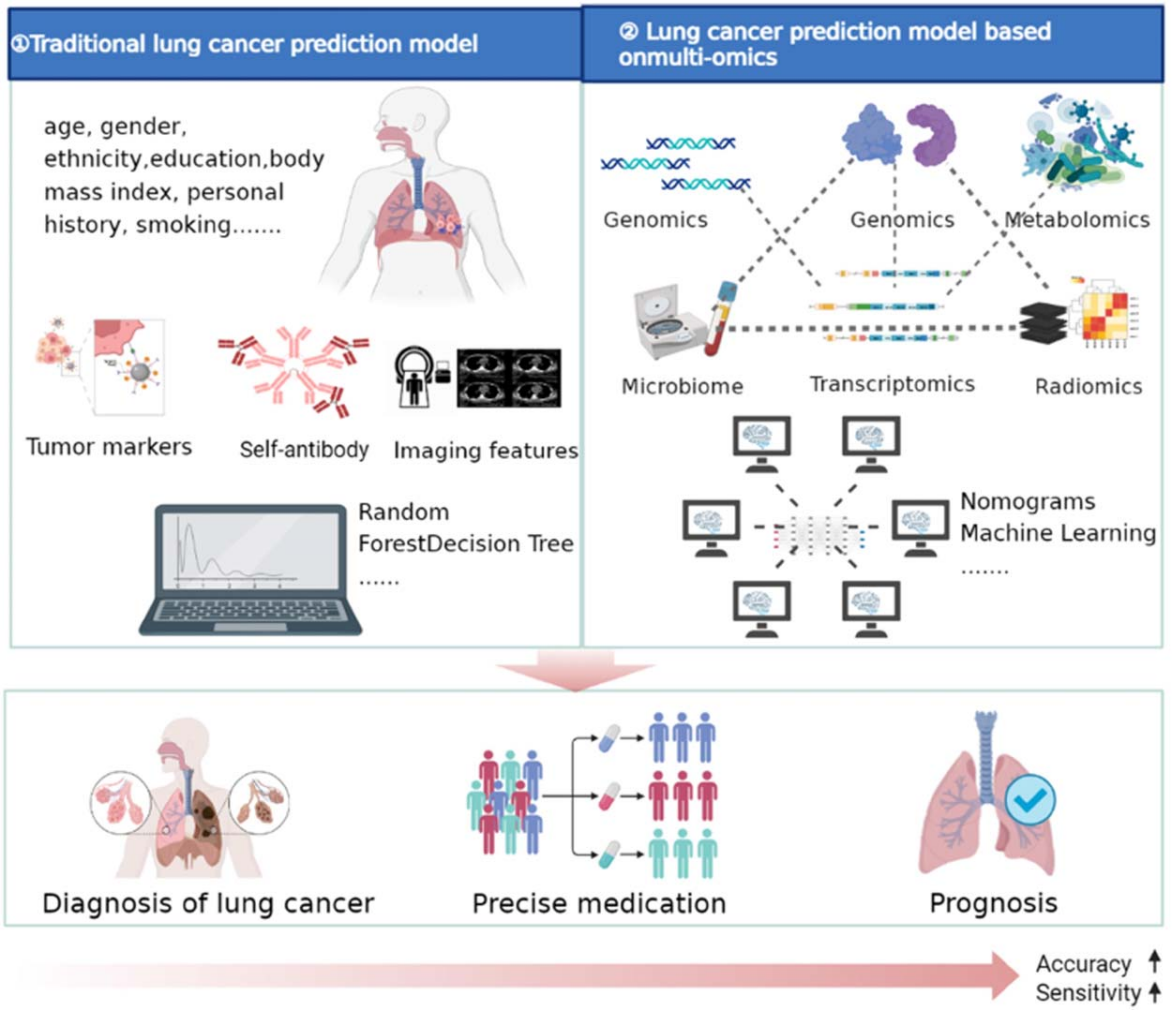
Evolution of lung cancer-predictive models.

Most such models consider age, sex, race, ethnicity, education, body mass index, personal history of cancer, personal history of pneumonia, family history of lung cancer, and various aspects of smoking exposure as risk predictors.^19,44^ In recent years, improvements in imaging, molecular biology, and omics research have led to many new diagnostic predictors (Fig. 10).^5^ A prediction model was constructed based on three DNA methylation biomarkers and one radiological characteristic, and achieved an area under the curve (AUC) value of 0.951 for malignant pulmonary nodule diagnosis, which was significantly higher than that of the Mayo Clinic model (AUC=0.823).^45^ Numerous studies have demonstrated that prediction models that consider biomarkers, radiomics, and genomics, and particularly models composed of multiple omics, could be conducive to understanding the underlying regulation of lung cancer growth and the distinction of benign and malignant lung nodules.^46–48^ The multi-omics integration analyses can be allowed for a deeper understanding of lung cancer onset and progression, the development of new treatment approaches and help to move the study of lung cancer from fundamental research to practical applications.^49,50^

Based on keyword analysis, we also found that nomograms (AAY: 2020.17) have become popular for lung cancer-prediction models in recent years, due to their accuracy and reliability. Machine learning (AAY: 2019.91) is a rapidly developing field of computational science and plays an important role in the construction of lung cancer-prediction model. Hosny *et al*.^51^ provided a new lung cancer-prediction model based on deep-learning network and CT images from patients with non-small cell lung cancer, it may be used for mortality risk stratification. Takahashi *et al*.^48^ used unsupervised machine learning techniques to build a model for lung cancer patient prognosis prediction, using six different multi-omics datasets from The Cancer Genome Atlas. The combination of multi-omics with machine learning technology is exceptionally promising in this area.

Top-cited references typically focused on epidemiological and lung cancer-prediction models. The most frequent co-cited reference was “*Reduced lung-cancer mortality with low-dose computed tomographic screening*,” published in *The New England Journal of Medicine* in 2011.^36^ The study of 53 454 high-risk persons at 33 US medical centers showed that screening with LDCT reduced lung cancer-associated mortality. In 2013, the USA Preventive Services Taskforce recommended annual lung cancer screening with LDCT.^52^ LDCT screening can reduce lung cancer mortality but also increase unnecessary examination, overtreatment, the risk of anxiety and rarely radiation-induced cancers.^3^ Prediction models based on multi-omics decrease the physical and mental burden of patients and healthcare system overload, and optimize the precision of diagnosis.^53^ As lung cancer produces significant morbidity and the limitations of the existing screening techniques,^54^ lung cancer-prediction models based on multi-omics are urgently needed and are likely to continue developing in future.

### Challenges and perspectives

Although lung cancer-prediction models have progressed significantly over the past two decades, many challenges remain. First, some prediction models were based on data from single-center or small-sample retrospective studies and lacked external datasets, while some studies with relatively large sample sizes were performed in European and American countries. Some studies have highlighted the importance of creating novel prediction models rather than optimizing existing models, with unnecessary waste of medical resources. Second, sensitivity and precision varied among models due to the different algorithms and data sources. This has created serious difficulties in establishing a unified diagnostic criterion. Third, some guidelines recommend the use of predictive models for lung cancer screening to decrease the false-positive rate of LDCT; however, no predictive model is currently widely accepted. Lung cancer-prediction models based on multi-omics data are intensely researched at present; however, integration techniques for multi-omics data are in the early stages of development. Finally, collaboration among different countries, institutions, and authors is not optimal. We emphasize the urgent need to strengthen cross-institutional, cross-regional, and transnational collaboration.

In the future, large‐scale and global multicenter studies are therefore needed to increase diagnostic efficiency and universality of lung cancer-prediction models and to maximize clinical benefit. Researchers should pay more attention to quality control, integration of differing sources of multi-omics data, and external validation of prediction models to improve the external applicability of the models and accelerate the progress toward the era of precision medicine.

The study had the following limitations. First, we only used reviews and articles published in English, between 2002 and 2022, which could lead to selection bias. Second, although the majority of publications included in the WoSCC database are of high quality, this inevitably leads to bibliography omissions. Third, many authors share the same first name and last name with other authors, and bibliometric software cannot distinguish the contributions of authors with the same name, making it difficult to avoid inaccuracies in the authors’ information.

## Conclusions

Lung cancer-prediction models are at a highly developed stage and have great clinical application potential. Over the past two decades, countries with strong scientific creativity have emerged, including the USA and China. The institutions represented by *Fudan University* have a significant academic influence in the field. John K Field and Jie He published the most reports. Prognosis, machine learning, and multi-omics technologies are the focus of current and future research and have shown great promise for applications. Finally, to enhance the clinical utility of prediction models, we recommend the use of external validation using data from large multicenter studies and increased collaboration across countries.

## Ethical approval

Not applicable.

## Consent

Not applicable.

## Source of funding

This work was supported by grants from the Postdoctoral Fellowship Program of CPSF under Grant Number GZC20230339 and the Science and Technology Department of Sichuan Province (2022ZDZX0022).

## Author contribution

Q.M. and H.J.: wrote the manuscript and data interpretation; S.-Y.T. and Q.W.: collected the data and performed the data analysis; F.-M.Y. and C.Z.: participated in the design of the study; Y.-F.R.: wrote the manuscript, data interpretation conceived and designed the study. All authors contributed to the review of the manuscript and approved the submitted version.

## Conflicts of interest disclosure

The authors declare no conflicts of interest.

## Research registration unique identifying number (UIN)

1. Name of the registry: not applicable.
2. Unique identifying number or registration ID: not applicable.
3. Hyperlink to your specific registration (must be publicly accessible and will be checked): not applicable.

## Guarantor

Yi-Feng Ren.

## Data availability statement

The data in this review are not sensitive in nature and is accessible in the public domain. The data are therefore available and not of a confidential nature. Additional data are made available in supplementary material of this manuscript.

## Provenance and peer review

Not commissioned, externally peer-reviewed.

## Supplementary Material
