# Supplementary material for: Emerging trends and hotspots in lung cancer-prediction models research
Source: Ann Med Surg (Lond). 2024 Oct 18;86(12):7178–92. doi: 10.1097/MS9.0000000000002648 (PMC11623829; doi:10.1097/MS9.0000000000002648)
Supplement: Supplementary file 1 [file ms9-86-7178-s001.docx]

**Emerging trends and hotspots in Lung Cancer Prediction Models research**

**Supplementary**

| **Table of Contents** | |
| --- | --- |
| **Table S1:** Web of Science Core Collection Search Strategy | **Page 2** |

**Table S1: Web of Science Core Collection Search Strategy (4,816)**


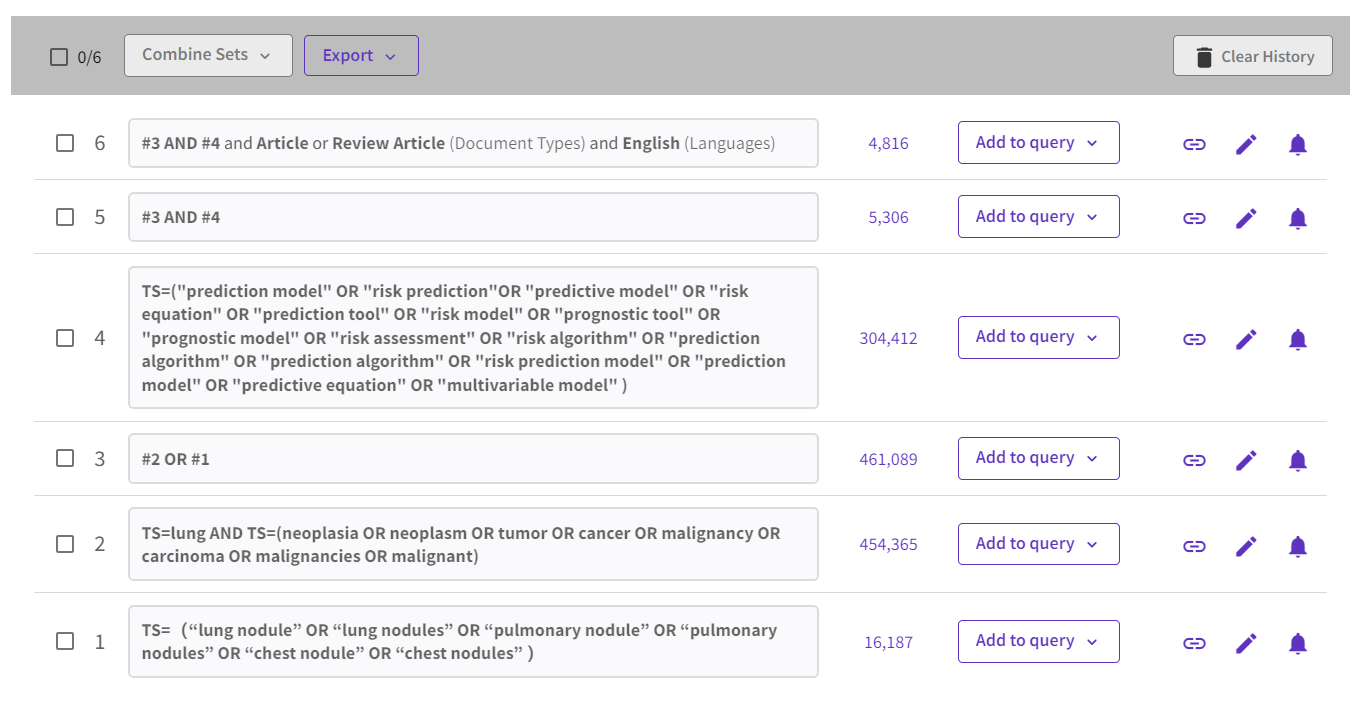


1. **TS=（“lung nodule” OR “lung nodules” OR “pulmonary nodule” OR “pulmonary nodules” OR “chest nodule” OR “chest nodules” ）** [16,187](https://ersp.lib.whu.edu.cn/s/com/webofscience/www/G.https/wos/woscc/summary/1792c6b5-d60c-4a62-8902-a413988aaf59-5f922437/relevance/1)
2. **TS=lung AND TS=(neoplasia OR neoplasm OR tumor OR cancer OR malignancy OR carcinoma OR malignancies OR malignant)** [454,365](https://ersp.lib.whu.edu.cn/s/com/webofscience/www/G.https/wos/woscc/summary/aebefcaf-7bce-45c4-9ee2-a44aeaf468d9-5f922999/relevance/1)
3. **#2 OR #1** [461,089](https://ersp.lib.whu.edu.cn/s/com/webofscience/www/G.https/wos/woscc/summary/1eb36fac-6582-46c4-bb4c-65893413eca1-5f9230aa/relevance/1)
4. **TS=("prediction model" OR "risk prediction"OR "predictive model" OR "risk equation" OR "prediction tool" OR "risk model" OR "prognostic tool" OR "prognostic model" OR "risk assessment" OR "risk algorithm" OR "prediction algorithm" OR "prediction algorithm" OR "risk prediction model" OR "prediction model" OR "predictive equation" OR "multivariable model" )** [304,412](https://ersp.lib.whu.edu.cn/s/com/webofscience/www/G.https/wos/woscc/summary/221b04bc-9cf4-409b-abdf-0527eb9b52ca-5f923244/relevance/1)
5. **#3 AND #4** [5,306](https://ersp.lib.whu.edu.cn/s/com/webofscience/www/G.https/wos/woscc/summary/6e264483-ae3c-4814-bbca-3d1f3457c038-5f925b1d/relevance/1)
6. **#3 AND #4** and **Article** or **Review Article** (Document Types) and **English** (Languages) 4816
